# An international qualitative study of functioning in autism spectrum disorder using the World Health Organization international classification of functioning, disability and health framework

**DOI:** 10.1002/aur.1905

**Published:** 2017-12-11

**Authors:** Soheil Mahdi, Marisa Viljoen, Tamara Yee, Melissa Selb, Nidhi Singhal, Omar Almodayfer, Mats Granlund, Petrus J. de Vries, Lonnie Zwaigenbaum, Sven Bölte

**Affiliations:** ^1^ Center of Neurodevelopmental Disorders (KIND), Division of Neuropsychiatry, Department of Women's and Children's Health Karolinska Institutet Stockholm Sweden; ^2^ Center for Psychiatry Research Stockholm County Council Stockholm Sweden; ^3^ Division of Child & Adolescent Psychiatry University of Cape Town Cape Town South Africa; ^4^ Department of Pediatrics University of Alberta Edmonton Canada; ^5^ ICF Research Branch, a cooperation partner within the WHO Collaborating Centre for the Family of International Classifications in Germany (at DIMDI) Nottwil Switzerland; ^6^ Swiss Paraplegic Research Nottwil Switzerland; ^7^ Action for Autism, The National Centre for Autism New Delhi India; ^8^ Mental Health Department KAMC‐R, MNGHA Riyadh Saudi Arabia; ^9^ CHILD, SIDR, School of Health and Welfare Jönköping University Jönköping Sweden

**Keywords:** autism spectrum disorder, functioning, strength, assessment, clinical practice, ICF, qualitative study

## Abstract

This is the third in a series of four empirical studies designed to develop International Classification of Functioning, Disability and Health (ICF) Core Sets for Autism Spectrum Disorder (ASD). The present study aimed to describe functioning in ASD (as operationalized by the ICF) derived from the perspectives of diagnosed individuals, family members, and professionals. A qualitative study using focus groups and semi‐structured interviews were conducted with 19 stakeholder groups (*N* = 90) from Canada, India, Saudi Arabia, South Africa, and Sweden. Meaningful concepts from the focus groups and individual interviews were linked to ICF categories using a deductive qualitative approach with standardized linking procedures. The deductive qualitative content analysis yielded meaningful functioning concepts that were linked to 110 ICF categories across all four ICF components. Broad variation of environmental factors and activities and participation categories were identified in this study, while body functions consisted mainly of mental functions. Body structures were sparsely mentioned by the participants. Positive aspects of ASD included honesty, attention to detail, and memory. The experiences provided by international stakeholders support the need to understand individuals with ASD in a broader perspective, extending beyond diagnostic criteria into many areas of functioning and environmental domains. This study is part of a larger systematic effort that will provide the basis to define ICF Core Sets for ASD, from which assessment tools can be generated for use in clinical practice, research, and health care policy making. ***Autism Res*** *2018, 11: 463–475*. © 2017 The Authors Autism Research published by International Society for Autism Research and Wiley Periodicals, Inc.

**Lay Summary:**

The study findings support the need to understand the living experiences of individuals with Autism Spectrum Disorder (ASD) from a broader perspective, taking into account many areas of an individual's functioning and environment. The ICF can serve as foundation for exploring these living experiences more extensively by offering tools that enable wide variety of individual difficulties and strengths to be captured along with important environmental influences. As such, these tools can facilitate interventions that meet the needs and goals of the individual.

## Background

Autism Spectrum Disorder (ASD) is a neurodevelopmental condition defined by persistent difficulties in social communication and interaction alongside stereotyped and repetitive behavior patterns [APA, 2013]. ASD is associated with a wide range of impairments, ranging from physical [Cashin, Buckley, Trollor, & Lennox, 2016] and psychiatric complaints [Simonoff et al., 2008] to significant interferences with occupational [Howlin, Moss, Savage, & Rutter, 2013], educational [Levy & Perry, 2011] and social life [Schmidt et al., 2015]. ASD has also been reported to be accompanied by specific strengths, such as attention to detail [Baron‐Cohen, Ashwin, Ashwin, Tavassoli, & Chakrabarti, 2009], visuo‐spatial skills [Happé & Frith, 2009], and increased auditory perceptual capacity that enables individuals to sustain performance on tasks requiring selective attention [Remington & Fairnie, 2017]. While a diagnosis of ASD requires evidence of functional impairment, the individual profile of functioning across life domains might differ substantially depending on developmental level and context. Therefore, the availability of internationally accepted, standardized classification tools for individual assessment of functional ability and disability of those living with ASD is desirable for clinical and educational settings, for research, and in healthcare administration. In 2001, the World Health Organization (WHO) endorsed the International Classification of Functioning, Disability and Health (ICF) to serve as a global standard for developing such tools by offering a comprehensive, unified framework to describe functioning in all health‐related conditions [WHO, 2001].

**Figure 1:**
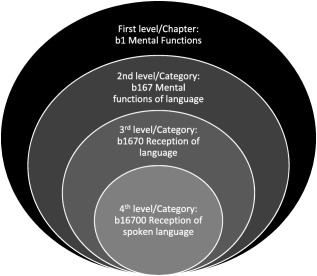
Example of the hierarchically organized category structure of the ICF.

The ICF is based on a bio‐psycho‐social framework, which sees an individual's abilities and disabilities as a result of the interplay between a health condition, environmental, and personal factors [WHO, 2001, 2007]. Designed to assess functioning on a continuum beyond diagnosis, the ICF complements the International Statistical Classification of Diseases and Related Health Problems‐Tenth Revision (ICD‐10), which primarily defines specific health conditions in a categorical fashion [Kostanjsek, 2011; WHO, 2001]. An integral part of the diagnostic process in the upcoming ICD‐11 will include using the categories from the ICF to capture the impact of health condition on functioning [WHO, 2014]. The ICF [WHO, 2001, 2007] comprises of 1,685 categories distributed across four components of health independent of diagnoses: body functions (*k* = 531), body structures (*k* = 329), activities and participation (*k* = 552), and environmental factors (*k* = 273). For each of these components, aspects of functioning are described in four different levels of depth as demonstrated by the following body function component example in Fig. 1.

The ICF framework also includes personal factors that are inherent to the individual but not part of the individual's primary health condition, such as gender, race, educational level, coping strategies etc. Personal factors are not yet classified in the ICF given their broad social and cultural variability [WHO, 2001, 2007].

The ICF provides a comprehensive, common language for clinical, public health, and research applications to facilitate documentation and measurement of health and functioning across the lifespan for diagnostic, treatment, and reimbursement purposes [Bölte, 2009; Escorpizo et al., 2013]. Using all the ICF categories to characterize an individual with a specific health condition would, however, be unnecessary, time‐consuming, and impractical, as many categories are irrelevant to specific conditions. To ensure the practical application of the ICF in various settings, including clinical practice, the development of ICF Core Sets was initiated by providing shortlists of categories that are relevant to specific health conditions [Selb et al., 2015]. The development of ICF Core Sets comprises four preparatory studies, namely a qualitative study, a literature review, an expert survey, and a clinical study. Each study aims to capture general and unique features of functioning specific to a certain health condition, ensuring that the process includes a wide range of professionals and stakeholders across all of the WHO‐regions. The present study is, therefore, part of a larger systematic effort that will subsequently lead to the development of standardized ICF Core Sets for ASD [Bölte et al., 2014].

The objective of the current study was to explore client and caregiver perspectives on functioning and environment pertinent to ASD as described by the ICF. In order to compare findings with results from the previous preparatory studies [de Schipper et al., 2015, 2016] within the ASD Core Set development, an exploratory secondary objective was added to also determine the consistency of identified ICF concepts by translating verbal material to numerical material (number of times a certain ICF category was mentioned). For this purpose, a qualitative and mixed methodology study as outlined by the WHO and ICF Research Branch [Selb et al., 2015] was conducted, involving focus group discussions and individual semi‐structured interviews with clients, caregivers, and professionals.

## Methods

### Design and Procedure

The study was approved by the regional ethics review board in Stockholm and by local ethics review boards at other participating sites. Written and verbal consent was obtained from each participant and/or parent or legal guardian prior to study participation. The consent form assured voluntary study participation and confidentiality of the participants. Moreover, the consent form also included information about the focus groups and individual interviews being audio‐recorded. Although written legal guardian consent was mandatory for younger participants, the researchers also comprehensively informed the younger individuals about the purpose of the study and awaited their verbal approval before initiating the interviews. Participants were also fully informed about their rights to withdraw at any time without disadvantages and get information about stored data on them. A qualitative methodology was applied, combining focus group discussions and semi‐structured individual interviews for data collection. In order to explore different perspectives, 90 participants were divided into 19 groups (Table 1) according to age (child, adolescent, adult), informant (individual with ASD, family member, school personnel, professional caregiver, and interest organization), and WHO‐country (region). Participating countries included Canada (The Americas), India (South‐East Asia), Saudi Arabia (Eastern Mediterranean), South Africa (Africa), and Sweden (Europe). In previous preparatory qualitative studies [Boonen, van Berkel, Cieza, Stucki, & van der Heijde, 2009; Coenen, Basedow‐Rajwich, Konig, Kesselring, & Cieza, 2011; Gradinger et al., 2011, 2014], four to six focus groups had been sufficient to achieve data saturation. This study included 13 focus groups plus additional 26 individual interviews to meet the basic requirements for including an international sample as outlined by the WHO and ICF Research Branch [Selb et al., 2015]. Of the 19 stakeholder groups that were included in this study, 13 were conducted as focus groups.

**Table 1:** Composition of Stakeholder Groups by Country

Country	WHO‐region	Number of participants (%)	Data collection method (Focus groups or interviews)
**Canada**	**The Americas**	**14 (16%)**	**Both**
Adult with ASD^a^		1 (7%)	Semi‐structured interview
Professional caregivers		4 (29%)	Focus group
Family members		9 (64%)	Focus group
**India**	**South East Asia**	**21 (23%)**	**Focus groups**
Adults with ASD		4 (19%)	Focus group
Parents		5 (23%)	Focus group
School personnel		6 (29%)	Focus group
Trainee parents		6 (29%)	Focus group
**Saudi Arabia**	**Eastern Mediterranean**	**10 (11%)**	**Focus groups**
Parents		6 (60%)	Focus group
Health professionals		4 (40%)	Focus group
**South Africa**	**Africa**	**12 (13%)**	**Focus groups**
Adults with ASD		2 (17%)	Focus group
Family members		6 (50%)	Focus group
Mixed family/teachers		4 (33%)	Focus group
**Sweden**	**Europe**	**33 (37%)**	**Both**
Adolescents with ASD		4 (12%)	Semi‐structured interviews
Adults with ASD		4 (12%)	Semi‐structured interviews
Children with ASD		4 (12%)	Semi‐structured interviews
Interest organization members		4 (12%)	Focus group
Parents to children		5 (16%)	Semi‐structured interviews
Parents to adolescents		4 (12%)	Focus group
Professional caregivers		4 (12%)	Semi‐structured interviews
School personnel		4 (12%)	Semi‐structured interviews

a Since only one adult was included from Canada, this adult was grouped into the Swedish adult stakeholder group for the frequency analysis.

The adequate number of participants for focus group discussions is based on topic complexity, with six to ten participants usually viewed as optimal [Morgan, 1998]. However, for the focus group discussions in the current study, we were mindful of the ASD‐related difficulties in social interaction and communication that might interfere with focus group discussions. For this reason, smaller groups of four participants were judged more appropriate to facilitate communication between individuals diagnosed with ASD and focus group moderators. Groups that involved other stakeholders were larger and included four to nine participants. To accommodate logistical challenges, which included last minute cancellations of scheduled focus groups, and to comply with participants’ preferences to participate in more intimate and anonymous interviewing, semi‐structured interviews were also conducted. The focus groups generally lasted between 35 and 94 min (Md = 66), while the individual interviews took 15–115 min (Md = 38) to complete. The focus groups and individual interviews were led by a moderator, either a clinician or clinical researcher experienced in ASD. The group discussions and individual interviews were audio‐recorded and transcribed verbatim. Transcriptions were then translated into English by approved translators.

### Participants

In total, 102 participants who fulfilled criteria for participation were contacted for the study between February and December 2015. Inclusion criteria for participants were either (a) having a primary clinical diagnosis of ASD or an ASD subtype according to the diagnostic criteria of the ICD‐10, DSM‐IV/‐TR, or DSM‐5 and/or receiving treatment for ASD; or (b) being an immediate family member, professional caregiver, school personnel, or other closely involved in the everyday life of individuals with ASD. Participants were excluded from the study if they were younger than 7 years of age or could not communicate in the language of the country where the focus group or interview took place. Recruitment of participants was mainly made via clinical research teams in the respective country and via invitations in collaboration with local and national interest organizations for ASD. Some participants were recruited from previous or still ongoing projects in the clinical research departments. The contributions of participants in respective countries were made by members of the project Steering Committee (see acknowledgment), a group of ASD experts, consisting of researchers, educators, clinicians and self‐advocates from all six WHO‐regions. The experts monitored the study progress and assisted in the practical elements of the study, such as finding clinicians and clinical researchers worldwide who could recruit participants and conduct the focus groups/individual interviews.

### Material

The focus groups and interviews were conducted using an interview guide comprising of six items covering all the ICF components, plus one item related to strengths associated with ASD (see Supporting Information Appendix 1). For the younger participants diagnosed with ASD, the interview started with easier questions related to activities in daily living, environmental factors, and individual strengths. Body functions and body structures were discussed later in the interviews/focus groups, with the moderator using nonverbal communication and hand gestures (e.g., pointing to the heart or the brain) to further communicate the intent of the questions. Some clinicians who knew the younger participants from previous encounters (e.g., assessments, interventions) asked follow‐up questions and encouraged the participants to discuss daily life issues that he or she had experienced. To avoid asking leading questions that might prompt the participant to respond in a desired way, the moderators/interviewers were instructed to be mindful of the way they asked the questions, ensuring that the responses reflected the views of the participant and not the professional. Visual aids (e.g., drawings, photographs) were also used in some cases to communicate feelings or thoughts about certain topics. Some of the interviews were arranged in rooms that included whiteboards, which the participant could use to draw symbols or sketches to communicate feelings or thoughts on specific topics.

### Analysis of Verbal Material: Meaningful Concepts and ICF Linking

The transcripts from the focus groups and individual semi‐structured interviews were translated into English prior to the identification of meaningful concepts. A deductive qualitative content analysis [Krippendorff, 2013] was conducted to extract meaningful units from the verbatim transcripts. A meaningful unit within the ICF Core Set preparatory research does not follow linguistic grammatical rules, rather the text is divided where a shift in meaning is observed [Karlsson, 1995]. Based on the meaningful units, the researchers extracted concepts that were then linked to ICF categories following a set of formal rules and procedures as determined by the ICF Research Branch [Cieza et al., 2002, 2005]. “Meaningful concepts” refers in this context to concepts that reflect the essence of what statements are saying. The linking rules also include terms that describe a meaningful concept that cannot be linked to any specific ICF category. These include: (a) ‘personal factor’; (b) ‘not covered’: concept that is not captured in the ICF and nor is a personal factor; (c) ‘nondefinable’: the information provided in the concept is insufficient for assigning a specific ICF category; and (d) ‘health condition’, if the concept refers to a diagnosis or health condition. Recurring ASD‐related strengths mentioned by the participants were also analyzed and linked to ICF categories as stated above.

To ensure the consistency of linking results for each focus group and semi‐structured interview, both the identification of meaningful concepts and linking of ICF categories were conducted by two independent researchers. At least one independent researcher was from a study country (excluding India) in order to capture different cultural expressions that were contained in the participants’ responses. In total, seven independent researchers were involved in the linking. To prepare for the linking of actual data, the researchers received linking exercises and vignettes from the ICF Research Branch (http://www.icf-research-branch.org). After the researchers completed the linking of focus groups and semi‐structured interviews, they compared their results. Consensus discussions were used to resolve disagreements. Cohen's Kappa coefficients (*ĸ*) for the second level ICF categories in the focus group discussions and semi‐structured interviews were respectively *ĸ* = 0.60 (SE = 0.010) with a confidence interval of *ĸ* = 0.58–0.62, and *ĸ* = 0.73 (SE = 0.009) with a confidence interval of *ĸ* = 0.71–0.75. These indicate fair to good agreement, irrespective of data collection method applied.

### Consistency of Quoted ICF Categories

To examine the consistency of concepts that were extracted from the meaningful units in transcripts and linked to ICF categories, orienting frequency analyses were conducted on the transcriptions from the different stakeholder groups. In this study, ICF categories are presented at second‐level. If a concept was linked to a third‐ or fourth level ICF category, the corresponding second‐level category was reported. To avoid bias in favor of participants that repeatedly expressed similar statements or were prompted by other participants’ responses, an ICF category was only counted once for each stakeholder group that involved focus group discussions (max. 13) or individual semi‐structured interviews (max. 6). To fully use the material from the verbatim transcripts and maximize all input put forward by the participants, an ICF category that was mentioned at least once per stakeholder group was included in the list of candidate categories [Boonen et al., 2009; Coenen et al., 2011]. Strengths were analyzed by exploring the consistency of recurring ASD‐related abilities.

## Results

### Sample

Of the 102 participants who were eligible for study participation, 90 completed the focus group discussions or semi‐structured interviews. Attrition in 12 participants was due to no shows (*n* = 8), or declining to participate in the study (*n* = 4) after initial consent. Table 2 summarizes the characteristics of participants who were included in the final analysis with respect to stakeholder group membership, gender, and age. Younger individuals with ASD were only included from Sweden, while adults with ASD and health/school personnel working with the diagnosis group were either underrepresented or missing in some of the study sites (e.g., Saudi Arabia and South Africa). Table 3 presents the socio‐demographic background of diagnosed individuals included in the study.

**Table 2:** Characteristics of Study Participants

Stakeholder groups	Size of group N (%)	Gender (female/male) N (%)	Age M (SD) Range
**Clients**	**19 (21)**	**8/11** **(39/61)**	**25 (16.2)** **9–67**
Children	4 (21)	2/2 (50/50)	11 (1.5) 9–12
Adolescents	4 (21)	0/4 (100/0)	16 (1.8) 14–17
Adults	11 (58)	6/5 (55/45)	34 (16.2) 18–67
**Immediate family members** ^a^	**43 (48)**	**39/4** **(91/9)**	**43 (10.6)** **22–68**
Parents/grandparents	37 (79)	33/4 (89/11)	45 (10.2) 23–68
Trainee parents	6 (13)	6/0 (100/0)	32 (5.3) 22–37
**Professional caregivers** Interest organization members^b^	**28 (31)** 4 (14)	**22/6** **(79/21)** 3/1 (75/25)	**43 (12.9)** **23–73** 50 (11.6) 34–62
School personnel^c^	12 (43)	10/2 (83/17)	43 (13.9) 23–73
Other professionals^d^	12 (43)	9/3 (75/25)	40 (12.0) 24–59

a Immediate family members consisted of individuals who had family relatives diagnosed with ASD and interest organization members.

b Interest organization members consisted of individuals who had family relatives diagnosed with ASD. The members work with raising awareness about ASD and support those who have the diagnosis, as well as their relatives.

c School personnel included teachers, special educators and principals.

d Other professionals included health professionals (e.g., psychiatrists, psychologists etc.) and individuals who work closely with individuals with ASD in daily life, such as personal assistants and residential caregivers.

**Table 3:** Socio‐Demographic Background of Diagnosed Individuals Participating in the Study

	Children 9–12 years *N* (%)	Adolescents 13–17 years *N* (%)	Adults >18 years *N* (%)
**ASD subtype**			
Asperger syndrome	2 (50)	2 (50)	7 (64)
Classic autism/autistic disorder	1 (25)	1 (25)	
Atypical autism	1 (25)	1 (25)	
Did not report			4 (36)
**Co‐morbidity**			
Yes^a^	4 (100)	2 (50)	5 (45)
No	0	2 (50)	6 (55)
**Treatment**			
Treatment received^b^	2 (50)	2 (50)	4 (36)
No treatment	2 (50)	2 (50)	7 (64)
**Education background**			
Primary/high school	4 (100)	4 (100)	2 (18)
College/university			5 (46)
Vocational training			2 (18)
Did not report			2 (18)
**Living situation**			
Living with parents	4 (100)	3 (75)	5 (46)
Living with a partner			4 (36)
Living independently			2 (18)
Other unspecified living situation		1 (25)	
**Working status**			
Students	4 (100)	4 (100)	3 (27)
Full time employment			1 (9)
Self‐employment			1 (9)
Supported employment			3 (9)
Retired/volunteer work			1 (9)
Did not report			2 (18)

a Co‐morbidities included attention deficit hyperactivity disorder (ADHD), oppositional defiant disorder (ODD), obsessive compulsive disorder (OCD), developmental coordination disorder (DCD), depression, generalized anxiety disorder (GAD), Tourette syndrome, and Turner syndrome.

b Treatments included medication and psychosocial treatment (e.g., social skills training).

Of the 43 interviewed immediate family members, 13 reported to be related to adults with ASD (18+ years of age), 12 to young school‐age children (7–12 years of age), 11 to preschool children (3–6 years of age), and 6 to older school‐age children/adolescents (13–17 years of age). Most family members mentioned to be related to individuals diagnosed with Classic autism/Autistic Disorder (*n* = 19), followed by Asperger's syndrome (*n* = 17) and Atypical autism (*n* = 5). Two did not respond to this question. A large majority of the interviewed professionals mentioned to be working with individuals with ASD across the lifespan (*n* = 20), while few mentioned to be exclusively working with children (*n* = 3), preschool children (*n* = 2), adults (*n* = 2), and adolescents (*n* = 1). Most professionals worked with individuals across the entire spectrum (*n* = 22).

### Meaningful Concepts and ICF Linking

The analysis of the 19 stakeholder groups yielded a total of 4,146 meaningful concepts that were linked to 110 second‐level ICF categories, 492 personal factors (e.g., honesty, self‐esteem, age, sense of humor), 223 ‘not covered’ codes (e.g., bullying, crime, education programs for parents), 209 ‘nondefinable codes (e.g., understanding, body problems, structure), and 26 health condition codes (e.g., ADHD, depression, OCD). Data saturation [Bowen, 2008] showed that 20 second‐level ICF categories (18%) would have been missing if data was only based on sample from informants with ASD diagnosis, highlighting the importance of including other stakeholders. Additional analysis showed that two ICF categories (2%) would not have been identified if stakeholders only would have included families and diagnosed individuals. Nine ICF categories (8%) would have been excluded if results would have been based only on the Swedish study site. Further analysis showed that roughly 40% of the ICF categories that were found among children and adolescents were also covered in the adult population. Of the 60% that were not covered, a third were ICF categories that are rather specific to adulthood, such as those regarding domestic life (e.g., preparing meals, acquisition of services, and goods), major life areas (e.g., employment, economic transactions, economic self‐sufficiency), and social and civic life (e.g., community life).

In total, 110 ICF categories were found in the four ICF components: 45 activities and participation categories, 33 body functions, 29 environmental factors, and 3 body structures. Table 4 shows the second‐level categories that were identified in the activities and participation component and their consistency across stakeholder groups. Identified categories were spread across all of the nine chapters in this component. In one of the focus groups involving parents to preschool children, the topic of engagement in play was raised: *“She is crawling right now and he also starts to crawl behind her, follows her. He doesn't know how to play with her”*. In the same focus group, another participant discussed her child's discomfort during social encounters: *“Issues like my son, he doesn't like being touched. He doesn't like when someone touches him”*.

**Table 4:** Identified ICF Categories from the Activities and Participation Component and Consistency across Stakeholder Groups

Second‐level ICF category	Chapter‐level ICF category	N
d130 copying	d1 learning and applying knowledge	7
d132 acquiring information	d1 learning and applying knowledge	5
d161 directing attention	d1 learning and applying knowledge	4
d166 reading	d1 learning and applying knowledge	8
d172 calculating	d1 learning and applying knowledge	5
d177 making decisions	d1 learning and applying knowledge	5
d210 undertaking a single task	d2 general tasks and demands	10
d230 carrying out daily routine	d2 general tasks and demands	18
d240 handling stress and other psychological demands	d2 general tasks and demands	8
d250 managing one's own behaviour	d2 general tasks and demands	12
d310 communicating with –receiving –spoken messages	d3 communication	9
d315 communicating with –receiving –nonverbal messages	d3 communication	6
d330 speaking	d3 communication	12
d335 producing nonverbal messages	d3 communication	16
d345 writing messages	d3 communication	5
d350 conversation	d3 communication	8
d360 using communication devices and techniques	d3 communication	4
d440 fine hand use	d4 mobility	12
d446 fine foot use	d4 mobility	6
d455 moving around	d4 mobility	7
d470 using transportation	d4 mobility	7
d475 driving	d4 mobility	6
d510 washing oneself	d5 self‐care	8
d520 caring for body parts	d5 self‐care	11
d530 toileting	d5 self‐care	9
d540 dressing	d5 self‐care	10
d550 eating	d5 self‐care	9
d570 looking after one's health	d5 self‐care	12
d620 acquisition of goods and services	d6 domestic life	5
d630 preparing meals	d6 domestic life	1
d640 doing housework	d6 domestic life	4
d710 basic interpersonal interactions	d7 interpersonal interactions and relationships	16
d720 complex interpersonal interactions	d7 interpersonal interactions and relationships	18
d740 formal relationships	d7 interpersonal interactions and relationships	7
d750 informal social relationships	d7 interpersonal interactions and relationships	16
d760 family relationships	d7 interpersonal interactions and relationships	7
d820 school education	d8 major life areas	16
d845 acquiring, keeping and terminating a job	d8 major life areas	4
d850 remunerative employment	d8 major life areas	5
d860 basic economic transactions	d8 major life areas	5
d870 economic self‐sufficiency	d8 major life areas	1
d880 engagement in play	d8 major life areas	4
d910 community life	d9 community, social and civic life	5
d920 recreation and leisure	d9 community, social and civic life	17
d940 human rights	d9 community, social and civic life	1

*N* = Number of stakeholder groups that mentioned the ICF category.

Table 5 presents the second‐level categories that were covered in the body functions component and their consistency across stakeholder groups. A majority of the body function categories were mental functions (*k* = 18), but other aspects of the body were also covered, such as sensory, digestive, cardiovascular, and neuromusculoskeletal and movement‐related functions.

**Table 5:** Identified ICF Categories from the Body Functions Component and Consistency across Stakeholder Groups

Second‐level ICF category	Chapter‐level ICF category	*N*
b114 orientation functions	b1 mental functions	5
b117 intellectual functions	b1 mental functions	6
b122 global psychosocial functions	b1 mental functions	4
b125 dispositions and intra‐personal functions	b1 mental functions	15
b126 temperament and personality functions	b1 mental functions	14
b130 energy and drive functions	b1 mental functions	13
b134 sleep functions	b1 mental functions	12
b140 attention functions	b1 mental functions	12
b144 memory functions	b1 mental functions	14
b147 psychomotor functions	b1 mental functions	13
b152 emotional functions	b1 mental functions	15
b156 perceptual functions	b1 mental functions	8
b160 thought functions	b1 mental functions	10
b163 basic cognitive functions	b1 mental functions	7
b164 higher‐level cognitive functions	b1 mental functions	18
b167 mental functions of language	b1 mental functions	10
b172 calculation functions	b1 mental functions	3
b180 experience of self and time functions	b1 mental functions	4
b210 seeing functions	b2 sensory functions and pain	4
b230 hearing functions	b2 sensory functions and pain	11
b250 taste function	b2 sensory functions and pain	6
b255 smell function	b2 sensory functions and pain	5
b265 touch function	b2 sensory functions and pain	10
b270 sensory functions related to temperature and other stimuli	b2 sensory functions and pain	7
b280 sensation of pain	b2 sensory functions and pain	14
b455 exercise tolerance functions	b4 functions of the cardiovascular, hematological, immunological and respiratory systems	4
b510 ingestion functions	b5 functions of the digestive, metabolic and endocrine systems	4
b515 digestive functions	b5 functions of the digestive, metabolic and endocrine systems	7
b525 defecation functions	b5 functions of the digestive, metabolic and endocrine systems	4
b530 weight maintenance functions	b5 functions of the digestive, metabolic and endocrine systems	3
b760 control of voluntary movement functions	b7 neuromusculoskeletal and movement‐related functions	13
b765 involuntary movement functions	b7 neuromusculoskeletal and movement‐related functions	9
b770 gait pattern functions	b7 neuromusculoskeletal and movement‐related functions	6

*N* = Number of stakeholder groups that mentioned the ICF category.

Table 6 shows the frequencies of second‐level categories that were identified in the environmental factors component along with their consistency across stakeholder groups. Categories in this component were identified in all five chapters. Immediate family support was mentioned by one of the children in the study to be very important: *“My family helps me to understand what I should do and when I should do something. They help me to understand things that are difficult or easy for me to do”*, whereas another participant, an adolescent with ASD, discussed how overwhelming certain physical environments can be: *“When I am outside, in a crowded place with lots of sound and noises…I scream…and get annoyed. I try to run away from the noise as quickly as possible”*. An adult with ASD highlighted the significant effect positive attitudes can have on self‐understanding and awareness of own condition: *“With my family doctor I have been over time slowly educating her about challenges I face, and she's been very open to learning about them and to help me find resources that…will help me better understand who I am and thus help me to understand how do I live a positive, constructive way of life”*.

**Table 6:** Identified ICF Categories from the Environmental Factors Component and Consistency across Stakeholder Groups

Second‐level ICF category	Chapter‐level ICF category	*N*
e110 products or substances for personal consumption	e1 products and technology	6
e115 products and technology for personal use in daily living	e1 products and technology	15
e125 products and technology for communication	e1 products and technology	12
e130 products and technology for education	e1 products and technology	6
e240 light	e2 natural environment and human‐made changes to environment	6
e250 sound	e2 natural environment and human‐made changes to environment	14
e260 air quality	e2 natural environment and human‐made changes to environment	1
e310 immediate family	e3 support and relationships	17
e320 friends	e3 support and relationships	5
e325 acquaintances, peers, colleagues, neighbors and community members	e3 support and relationships	6
e330 people in positions of authority	e3 support and relationships	9
e340 personal care providers and personal assistants	e3 support and relationships	9
e355 health professionals	e3 support and relationships	8
e360 other professionals	e3 support and relationships	12
e410 individual attitudes of immediate family members	e4 attitudes	15
e415 individual attitudes of extended family members	e4 attitudes	1
e425 individual attitudes of acquaintances, peers, colleagues, neighbors and community members	e4 attitudes	5
e430 individual attitudes of people in positions of authority	e4 attitudes	7
e450 individual attitudes of health professionals	e4 attitudes	4
e455 individual attitudes of other professionals	e4 attitudes	5
e460 societal attitudes	e4 attitudes	8
e465 social norms, ideologies and practices	e4 attitudes	1
e550 legal services, systems and policies	e5 services, systems and policies	1
e560 media services, systems and policies	e5 services, systems and policies	3
e570 social security services, systems and policies	e5 services, systems and policies	2
e575 general social support services, systems and policies	e5 services, systems and policies	5
e580 health services, systems and policies	e5 services, systems and policies	8
e585 education and training services, systems and policies	e5 services, systems and policies	17
e590 labour and employment services, systems and policies	e5 services, systems and policies	5

*N* = Number of stakeholder groups that mentioned the ICF category.

Body structures component included three ICF categories, namely s110 structure of brain (*n* = 9; s1 structures of the nervous system), s320 structure of mouth (*n* = 4; s3 structures involved in voice and speech), and s750 structure of lower extremity (*n* = 4; s7 structures related to movement).

### ASD‐Related Strengths

In addition, certain strengths were captured in this study, as expressed by the following adult with ASD (b126 temperament and personality functions; *n* = 8): *“Honesty is something I like. If you do something, you should be able to stand for it. I am very frank with everything I do. I don't sugarcoat things. I often say what I think”*. A professional caregiver mentioned attention (b140 attention functions; *n* = 6) and out of box thinking (*n* = 5) to be positive aspects of ASD: *“Their intense focus. Their ability to maintain focus is a strong positive trait, as well as thinking outside of the box, which can be beneficial when they are faced with specific situations that require novel solutions”*. Other recurring themes and categories included b144 memory functions (*n* = 5), expertise in specific topics (*n* = 3), and d166 reading (*n* = 3).

## Discussion

This qualitative and mixed method study is one of four preparatory studies conducted within a superordinate project to develop WHO ICF Core Sets for ASD. We aimed to investigate the experiences and perspectives on functioning and environment pertinent to ASD put forward by diagnosed individuals with ASD and secondary informants living or working closely to them. Particularly, the study sought to highlight aspects of functioning and environment that these stakeholders considered to be important in the assessment of health‐related abilities and disabilities in ASD. Relevant functioning categories were found in all four ICF components. The activities and participation component and environmental factors were described comprehensively. Although a majority of the categories in the body functions component were mental functions, physical and sensory issues were also associated with ASD. Additionally, this study found information on specific strengths related to ASD, such as attention to detail, honesty and “out of the box thinking”.

### ICF Categories

The large number and variation of ICF categories captured in this study demonstrates the complex nature of ASD, as well as the importance of assessing many different areas of functioning in ASD. Apart from mental functions, physical (e.g., gastrointestinal problems) and sensory issues (e.g., sensation of pain, touch sensitivity) were also found to be affected, highlighting the broad impact ASD has on various body functions [Cashin et al., 2016; Marco, Hinkley, Hill, & Nagarajan, 2011; McElhanon, McCracken, Karpen, & Sharp, 2014]. As expected, the impact of ASD on everyday life activities was described comprehensively. Identified categories ranged from difficulties in management of everyday life demands and tasks to communication and social interaction problems. Other daily life domains included restrictions in leisure activities and school education. Similarly, all chapters from the environmental factors component were identified to be important in ASD, ranging from provision of education services and support from immediate family members to the use of different products and technology in daily life (e.g., cell‐phone, computer). The results here corroborate previous research findings, which show that environmental factors can play a key role in moderating ASD symptoms and facilitate better functional outcomes in individuals with ASD [Dawson et al., 2012; Kirby, Baranek, & Fox, 2016]. Contrary to our previous two preparatory studies [de Schipper et al., 2015, 2016], this study yielded a wider range of environmental factors, suggesting that contextual barriers and facilitators are more significant to functioning among caregivers and diagnosed individuals compared to the existing literature and expert opinion. A lack of emphasis on environmental factors has been pointed out previously for golden standard scales for diagnosing individuals with ASD [Castro, Ferreira, Dababnah, & Pinto, 2013]. Similar results were found when preschool curriculum content for individuals with special needs was linked to ICF categories [Castro, Pinto, & Maia, 2011]. The information on environmental factors is important, as it enables stakeholders to understand how individual abilities can be improved through facilitators and how barriers in the environment can be changed to fit the needs of those living with ASD. This qualitative study managed to show the importance of environmental factors by involving caregivers and diagnosed individuals. Compared to the other ICF components, few body structures were identified in this study. Although not formally linkable to ICF categories, a large number of personal factors, such as age, education level, living situation, were identified in this study, further attesting to the link between personal background experiences and the lived experiences of ASD [Delobel‐Ayoub et al., 2015; Durkin et al., 2010]. In combination with results from the other preparatory studies, the meaningful concepts that were not linked to ICF categories (i.e., personal factors, nondefined and noncovered codes) can be further examined in separate analyses that enable recurring themes to be derived [Finger, De Bie, Selb, & Escorpizo, 2016]. The meaningful concepts that were linked to personal factors will also in the future be further examined by linking these to a personal factors classification which has been proposed previously [Geyh et al., 2011]. Thus, the data on these meaningful concepts will not be lost, but subjected to further analyses that can potentially provide additional valuable information pertaining to functioning and environment in ASD.

### ASD‐Related Strengths

This study is amongst the first to explore specific strengths of individuals with ASD (as described by the ICF) using primary perspective from diagnosed individuals and perspectives from secondary informants living or working closely to individuals on the spectrum. The opinions of different stakeholders were generally rather broad and complex, reflecting both the clinical heterogeneity of the diagnosis, as well as diversity of lived experience. Some of our study findings are consistent with previous ASD research concerning systemizing and attention to detail [Baron‐Cohen, et al., 2009; Happé & Frith, 2009; Remington & Fairnie, 2017]. In addition, we found the present findings to be comparable with our previous preparatory expert survey [de Schipper et al., 2016]. Although previous research has identified circumscribed ASD‐related strengths, these have not been studied extensively regarding their relevance for daily living [Hillier et al., 2007]. For example, a study investigating the experiences of employees with ASD found that specific strengths were not fully utilized in the context of work, despite the individuals’ expertise and motivation to work [Baldwin, Costley, & Warren, 2014]. The underutilization of ASD‐related strengths in work environment can partly be related to the fact that more emphasis has been placed on exploring economic benefits with employing individuals with ASD rather than investigating individual strengths and resources that can benefit employers and colleagues [Jacob, Scott, Falkmer, & Falkmer, 2015; Scott, Falkmer, Girdler, & Falkmer, 2015]. Our findings on ASD‐related strengths can provide researchers with future means to develop tools (e.g., checklists, guidelines) that can be used by employment agencies or employers to facilitate successful job‐matching, by identifying not only potentially occupational challenges, but also strengths.

## Study Limitations

This study faces some methodological challenges. The generalizability of the consistency of recurring ICF categories across groups might be questioned, as the Western Pacific WHO‐region was not included. In addition, regarding the Americas WHO region, only North America was included in the study, limiting potential global generalization. It's important to remember that these orienting frequency analyses only reflect the consistency of ICF categories across groups and was mainly meant to facilitate comparisons with the other two previous preparatory studies [de Schipper et al., 2015, 2016]. Our saturation analyses showed identified categories to be quite exhaustive for ASD in general. The involvement of several culturally diverse countries also generated challenges concerning transcriptions into English. Proper translation of specific cultural expressions and their exact connotation were difficult, and in some cases impossible. While the linking was conducted in collaboration with a researcher located at one center for reasons of standardization and practicability, future studies might consider involving independent researchers to conduct the linking of ICF categories directly in their native language. Other significant weaknesses include the underrepresentation of some stakeholder groups in specific study sites (e.g., health/school personnel in South Africa) and the limited involvement of some subgroups of primary informants, more specifically children and adolescents, and intellectual disabled autistic individuals. Given that one of the important aims of the ICF Core Sets project is to involve perspectives of different stakeholders, larger number of younger individuals diagnosed with ASD should have been included in the study. However, the inclusion of four cross‐cultural studies in the ICF Core Sets development ensures that specific gaps in one study can be addressed by another study [Selb et al., 2015]. For example, the lack of children and adolescents with ASD will be addressed in the upcoming ICF clinical study, which involves multi‐international centers contributing with clinical cases of children and adolescents with ASD (both with and without intellectual disability). Part of the clinical study is to interview children and adolescents with ASD regarding different aspects of functioning and disability, ensuring that specific gaps in the current study are addressed in the upcoming preparatory study.

## Conclusions

This study identified a broad variety of ICF categories indicative of body functions/structure, activities and participation and environment significant to people living with ASD or close to people on the spectrum at different international research sites. This study also explored ASD‐related strengths. The current study is part of a larger systematic effort that involves three additional cross‐cultural investigations. Once the preparatory studies have been completed, the findings from the studies will be presented at an international consensus conference, involving a group of ASD experts representing various professional background and all WHO‐regions. Based on the results from the different preparatory studies, the experts will decide on which ICF candidate categories to include in the first version of the ASD ICF Core Sets. The development of ICF Core Sets for ASD will mark a milestone, as it will provide the basis for the development of user‐friendly, metrically‐sound assessment tools that cover the breadth of functional domains relevant to ASD. These tools will then be implemented and examined across different settings and age‐groups, outlining valid and reliable ways to assess functioning and disability in younger informants with ASD across the entire spectrum. A major advantage with these tools is that they will describe ASD‐related ability and disability beyond psychopathology and diagnostic information, taking into account other areas of functioning by integrating physical, mental and social aspects of the patient. Diagnoses are important for defining causes and prognosis, but limited in identifying concrete limitations or barriers in an individual's daily life, which is often required in order to plan individual‐based interventions or calculate individual health costs in societies. Being a member of the family of WHO classifications, tools derived from the Core Sets can be adapted to different cultural context and be applied all over the world. The common language used to describe functioning and disability in the ICF enables future tools that can improve communication between stakeholders across different groups and organizations, as well as generate future cross‐cultural comparisons of data.

## Conflict of interest

Soheil Mahdi, Marisa Viljoen, Tamara Germani, Melissa Selb, Nidhi Singhal, Omar Almodayfer, Mats Granlund, and Lonnie Zwaigenbaum declare no conflict of interest related to this work. Petrus J de Vries has no conflicts of interest related to this article. He has been on the study steering committee of three clinical trials sponsored by Novartis, is co‐PI on two investigator‐initiated studies part‐funded by Novartis, and is on the working group and scientific advisory board of a natural history study of tuberous sclerosis complex, sponsored by Novartis. Sven Bölte reports no direct conflict of interest related to this article. Bölte discloses that he has in the last 5 years acted as an author, consultant or lecturer for Shire, Medice, Roche, Eli Lilly, Prima Psychiatry, GLGroup, System Analytic, Kompetento, Expo Medica, and Prophase. He receives royalties for text books and diagnostic tools from Huber/Hogrefe, Kohlhammer and UTB.

## Supporting information

Supporting Information
