# Supplementary material for: An international qualitative study of functioning in autism spectrum disorder using the World Health Organization international classification of functioning, disability and health framework
Source: Autism Res. 2017 Dec 11;11(3):463–75. doi: 10.1002/aur.1905 (PMC5900830; doi:10.1002/aur.1905)
Supplement: Supplementary file 1 — Supporting Information [file AUR-11-463-s001.docx]

**Appendix 1.**


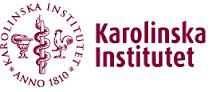

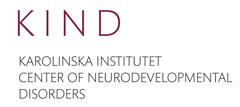


**Qualitative study on functioning and disability in ASD –client and caregiver perspective**

**1. How does your ASD affect the way your body and your mind works?** *(body functions)*

**2. In which parts of your body does your ASD give you problems?** *(body structures)*

**3. How does your ASD affect the things you can and cannot do you in your everyday life?** *(activities and participation)*

**4. What and/or who in the environment where you live and work/go to school make everyday life with ASD difficult for you?** *(environmental factors -barriers)*

**5. What and/or who in the environment where you live and work/go to school is helpful and supportive in your everyday life with ASD?** *(environmental factors –facilitators)*

**6. When you think about yourself and the person you are, what helps you to handle your everyday life with ASD?** *(personal factors)*

**7. What can be the positive sides of living with ASD?**
